# Differential Outcomes of Infection by Wild-Type SARS-CoV-2 and the B.1.617.2 and B.1.1.529 Variants of Concern in K18-hACE2 Transgenic Mice

**DOI:** 10.3390/v16010060

**Published:** 2023-12-29

**Authors:** Yicheng He, Jill Henley, Philip Sell, Lucio Comai

**Affiliations:** 1 Department of Molecular Microbiology and Immunology, 2011 Zonal Avenue, Los Angeles, CA 90089, USA; 2 Hastings Foundation and Wright Foundation BSL3 Laboratory, Keck School of Medicine, University of Southern California, Los Angeles, CA 90089, USA

**Keywords:** SARS-CoV-2, Delta and Omicron variants, infection, brain, cytokines, chemokines

## Abstract

Background: SARS-CoV-2 is a respiratory virus with neurological complications including the loss of smell and taste, headache, and confusion that can persist for months or longer. Severe neuronal cell damage has also been reported in some cases. The objective of this study was to compare the infectivity of the wild-type virus, Delta (B.1.617.2) and Omicron (B.1.1.529) variants in transgenic mice that express the human angiotensin-converting enzyme 2 (hACE2) receptor under the control of the keratin 18 promoter (K18) and characterize the progression of infection and inflammatory response in the lungs, brain, medulla oblongata, and olfactory bulbs of these animals. We hypothesized that wild type, Delta and Omicron differentially infect K18-hACE2 mice, thereby inducing distinct cellular responses. Methods: K18-hACE2 female mice were intranasally infected with wild-type, Delta, or Omicron variants and euthanized either at 3 days post-infection (dpi) or at the humane endpoint. None of the animals infected with the Omicron variant reached the humane endpoint and were euthanized at day 8 dpi. Virological and immunological analyses were performed in the lungs, brains, medulla oblongata and olfactory bulbs isolated from infected mice. Results: At 3 dpi, mice infected with wild type and Delta displayed significantly higher levels of viral RNA in the lungs than mice infected with Omicron, while in the brain, Delta and Omicron resulted in higher levels of viral RNA than with the wild type. Viral RNA was also detected in the medulla oblongata of mice infected by all these virus strains. At this time point, the mice infected with wild type and Delta displayed a marked upregulation of many inflammatory markers in the lungs. On the other hand, the upregulation of inflammatory markers was observed only in the brains of mice infected with Delta and Omicron. At the humane endpoint, we observed a significant increase in the levels of viral RNA in the lungs and brains of mice infected with wild type and Delta, which was accompanied by the elevated expression of many inflammatory markers. In contrast, mice which survived infection with the Omicron variant showed high levels of viral RNA and the upregulation of cytokine and chemokine expression only in the lungs at 8 dpi, suggesting that infection and inflammatory response by this variant is attenuated in the brain. Reduced RNA levels and the downregulation of inflammatory markers was also observed in the medulla oblongata and olfactory bulbs of mice infected with Omicron at 8 dpi as compared with mice infected with wild-type and Delta at the humane end point. Collectively, these data demonstrate that wild-type, Delta, and Omicron SARS-CoV-2 induce distinct levels of infection and inflammatory responses in K18-hACE2 mice. Notably, sustained brain infection accompanied by the upregulation of inflammatory markers is a critical outcome in mice infected with wild type and Delta but not Omicron.

## 1. Introduction

Coronavirus disease 2019 (COVID-19) is an infectious disease caused by severe acute respiratory syndrome coronavirus 2 (SARS-CoV-2) that has affected millions of individuals worldwide. SARS-CoV-2 is a β-coronavirus with a positive-sense RNA genome very similar to SARS-CoV-1 (80%), but more infectious and transmissive due to a higher reproductive number (R0) [1]. SARS-CoV-2 contains five viral proteins: a nucleocapsid (N) protein for coating and packaging the RNA genome, a membrane (M) protein for incorporating viral components into new virions, an envelope (E) protein for viral assembly, and the spike (S) protein contributing to viral entry into the host cells [2]. The first mutation found in SARS-CoV-2 was in the spike protein (D614G), which was then carried by many SARS-CoV-2 variants, including Delta (B.1.617.2) and Omicron (B.1.1.529) [3]. The D614G mutation results in increased infectivity by assembling more spike (S) proteins on the virions, which result in enhanced viral loads in the epithelial cells of the upper respiratory tract and lungs of COVID-19 patients [4,5].

Other mutations found in the Delta variant (B.1.617.2) have been implicated in the induction of a more severe disease. The Delta variant can fuse the cellular membrane more efficiently, infect target cells much faster, and has a higher virion-releasing advantage over other SARS-CoV-2 variants [6,7]. Some of the mutations on the spike protein responsible for these changes are T478K and P681R [8,9]. Different from other variants, Omicron (B.1.1.529) was found to be more dependent on the endocytic pathway for host-cell entry [10]. The Omicron variant has a large repertoire of mutations in the spike protein that can increase viral transmission and enhance cellular attachment [11,12,13]. However, studies have shown that the Omicron variant is compromised in the efficiency of viral fusion and viral replication competence [14,15], resulting in reduced viral load, attenuated inflammation, and overall severity of lung damage [16,17].

SARS-CoV-2 is a respiratory virus that can cause acute respiratory distress syndrome as well as neurological complications [18]. Patients being hospitalized due to acute infection have reported distinct neurological symptoms, including agitation, confusion, headache, and impaired consciousness [12,19]. Severe neuronal damage was observed in the post-mortem brains from patients who died of SARS-CoV-2. Neurological damage included hemorrhage with infarction and white matter lesions, venous thrombosis [20,21] or thrombotic ischemic infarction [21,22], ischemic necrosis [23], edema [24], perivascular or microvascular congestion and injury [23], and hypoxic alterations [25,26,27]. Cerebrospinal fluid (CSF) samples collected from patients with moderate to severe COVID-19 showed elevated cytokine levels [28,29]. However, since very few clinical samples detected the presence of viral RNA and viral proteins [30], these neurological complications did not appear to be caused by direct viral infection of the brain [20,21,22,23,24,25,26,27,28]. However, several laboratories have shown that SARS-CoV-2 can infect the brain and induce neuroinflammation by crossing the bloodbrain barrier [31]. Consistent with this finding, it has also been reported that the S1 subunit of the S protein can cross the blood–brain barrier and activate microglial cells to increase cytokine release and inflammasome activities [32,33,34]. Moreover, a study has shown that infection of human neural progenitor cells results in enhanced expression of viral transcripts and metabolic processes, highlighting the ability of SARS-CoV-2 to hijack the host neurons to replicate [35]. SARS-CoV-2 can also infect non-neuronal cells, inducing the upregulation of genes related to pro-inflammatory response and endoplasmic reticulum stress response (ER) [36]. In this study, to compare the infectivity of wildtype, Delta, and Omicron and examine the immune response in the lungs and brain of an animal model, we performed infections by these three strains of SARS-CoV-2 in the well-characterized K18-hACE2 transgenic mouse model.

## 2. Materials and Methods

### 2.1. Mice and Viruses

All animal procedures were performed at the Hastings Foundation and the Wright Foundation BSL3 facility of the Keck School of Medicine at the University of Southern California (USC) and were approved by the Institutional Animal Care and Use Committee (IACUC) and the Institutional Biosafety Committee (IBC) of USC.

K18-hACE2 transgenic mice were obtained from the Jackson Laboratory (strain #: 034860). Female mice between the ages of 8 and 10 weeks old were intranasally inoculated with SARS-CoV-2 and variants at 10^4^ PFU and euthanized at set end point of 3 dpi or the humane endpoint or 8 dpi which ever came first.

The wild-type, Delta and Omicron SARS-CoV-2, all noted as variants of concern (VOC) by the United States’ Center for Disease Control and Prevention (CDC) were obtained from the BEI repository: wild type (USA-WA1/2020, catalog: NR-52281), Delta (B.1.617.2, catalog: NR-55671), and Omicron (B.1.1.529, catalog: NR-56461). All virions were propagated and titered in Vero E6 cells overexpressing human ACE2 (VeroE6-hACE2) obtained from Dr. Jae Jung. 

### 2.2. Measurement of Viral Burden

Total RNA was collected from lungs, brains, olfactory bulbs and medulla oblongata of infected mouse tissues at the indicated time points using TRIzol reagent (Thermo Fisher Scientific, Waltham, MA, USA) according to the manufacturer’s instructions. Complementary DNA (cDNA) was synthesized from DNase-treated RNA using iScript Reverse Transcription Supermix for RT-qPCR (BIO-RAD catalog: 1708841). A sample of 100 ng of RNA template in a 10 µL volume reaction was used with 2 µL of iScript RT Supermix. Reaction protocol for cDNA synthesis was as follows: priming 5 min at 25 °C, reverse transcription 20 min at 46 °C, RT inactivation 1 min at 95 °C. Copies of the SARS-CoV-2 nucleocapsid (N1) gene were determined using IDT Primetime Gene Expression Master mix (Cat#1055770). The 2019-nCoV_N1 primer set was purchased from IDT (Cat#10007007). The primer sequence of N1 gene: Forward Primer: 5′-GAC CCC AAA ATC AGC GAA AT-3′ Reverse Primer: 5′-TCT GGT TAC TGC CAG TTG AAT CTG-3′ Probe: 5′-FAM-ACC CCG CAT TAC GTT TGG TGG ACC BHQ1-3′. Murine actin B (mActB) control primers were purchased from IDT (Mm.PT. 39a.22214843.g Cat# 1077852). A measured 10 µL volume reaction was used with 5 µL Primetime Gene Expression Master mix (2×), 1 µL cDNA template, 0.75 µL for N1 primer probe or 1 µL for mAct B primer-probe (final reaction concentration: 500 nM). Reaction protocol for RT-qPCR was as follows: primer melting for 15 min at 50 °C, pre-denaturing for 2 min at 95 °C, denaturation 3 s at 95, annealing 30 s at 55 °C (40 cycles). Known genomic equivalent standards for SARS-CoV-2 quantification were run simultaneously.

### 2.3. Measurement of Cytokines and Chemokines mRNA

RNAs from the lungs, brains, olfactory bulbs and medulla oblongata were extracted using the kit mentioned above. Complementary DNA (cDNA) was synthesized from DNase-treated RNA using iScript Reverse Transcription Supermix for RT-qPCR (BIO-RAD catalog: 1708841). A measured 20 µL volume reaction was used with 4 µL of iScript RT Supermix and 200 ng RNA template input. The protocol for cDNA synthesis was as follows: priming 5 min at 25 °C, reverse transcription 20 min at 46 °C, RT inactivation 1 min at 95 °C. Cytokine and chemokine expression were determined using SsoAdvanced Universal SYBR Green Supermix (BIO-RAD catalog: 1725271). The 10 µL volume reaction was used with 5 µL SsoAdvanced Universal SYBR Green Supermix (2×), 1 µL cDNA template, and 0.5 µL each forward and reverse primer (final reaction concentration: 500 nM). Reaction protocol RT-qPCR (SYBR Green) was as follows: initial DNA denaturing for 30 s at 95 °C, denaturing for 15 s at 95 °C, annealing/extension for 30 s at 60 °C (40 cycles). The melt curve was run after the completion of qPCR to assess the production of single products. Primer sets were:

CXCL9 (F: CCTAGTGATAAGGAATGCACGATG; R: CTAGGCAGGTTTGATCTCCGTTC); CXCL10 (F: ATCATCCCTGCGAGCCTATCCT; R: GACCTTTTTTGGCTAAACGCTTTC); CCL8 (F: GGGTGCTGAAAAGCTACGAGAG; R: GGATCTCCATGTACTCACTGACC); VEGF-α (F: GCACTGGACCCTGGCTTTAC; R: ATCGGACGGCAGTAGCTTCG); CCL2 (F: TGTTCACAGTTGCCGGCTG; R: GCACAGACCTCTCTCTTGAGC); IFN-γ (F: CAGCAACAGCAAGGCGAAAAAGG; R: TTTCCGCTTCCTGAGGCTGGAT); TNF-α (F: GGTGCCTATGTCTCAGCCTCTT; R: GCCATAGAACTGATGAGAGGGAG); CXCL11 (F: CCGAGTAAGGCTGCGACAAAG; R: CCTGCATTATGAGGCGAGCTTG); IL-6 (F: ACCCCAATTTCCAATGCTCTCCT; R: ACGCACTAGGTTTGCCGAGTA); IL-1β (F: TGGACCTTCCAGGATGAGGACA; R: GTTCATCTCGGAGCCTGTAGTG); IL-10 (F: CGGGAAGACAATAACTGCACCC; R: CGGTTAGCAGTATGTTGTCCAGC); GM-CSF (F: CCTGGGCATTGTGGTCTACAG; R: GGCATGTCATCCAGGAGGTT); G-CSF (F: GCAGACACAGTGCCTAAGCCA; R: CATCCAGCTGAAGCAAGTCCA)

### 2.4. Immunohistochemistry

Lung and brain tissue from mice were fixed in 10% formalin, embedded in paraffin, and sectioned (4–5 μm thickness). Tissue slides were baked at 60 °C for 30 min, deparaffinized with xylene and rehydrated using ethanol. Slides were then boiled for 20 min in antigen retrieval buffer (Tris-EDTA buffer, pH 9.0), and non-specific binding sites were blocked using 0.3% H_2_O_2_ as well as protein blocking reagent for 5 min. After blocking, the sections were incubated with SARS-CoV-2 antibody against the nucleocapsid (N) protein (Thermo Fisher MA536086; 1:28,000 dilution) for 15 min at room temperature. After washing, the slides were incubated with the secondary antibody (Abcam ab64261) for 8 min. Next, the chromogen DAB was applied to the slides for 5 min and slides were counterstained using hematoxylin for 10 min [35]. The slides were viewed in the bright field at 4× and 10× magnification on a Keyence microscope.

## 3. Results

### 3.1. Disease Progression of SARS-CoV-2 Infection of K18-hACE2 Mice

**Figure 1:**
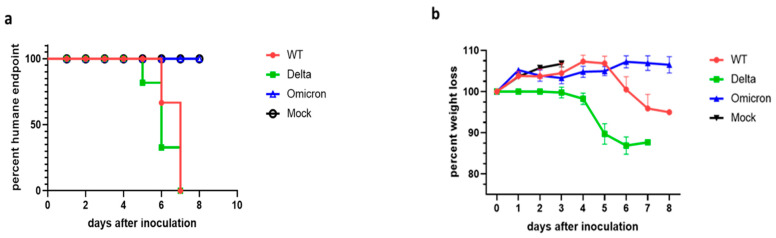
Probability of survival and body weight of SARS-CoV-2 infected K18-hACE2 mice. (**a**) Mice were infected at 10^4^ PFU with the indicated SARS-CoV-2 virus (mock = uninfected; WT = wild type). The panel shows the percentage of mice that reached humane endpoint in days after inoculation (a.k.a. infection) (*n* = 6). Mice infected with Omicron did not reach the humane endpoint and were euthanized at 8 days post infection (dpi). (**b**) Weight loss of uninfected mice (mock) and mice infected with wild type, Delta, and Omicron (*n* = 6). Mean  ±  s.e.m. Note: uninfected mousee weight is only shown over 3 days.

K18-ACE2 transgenic mice were infected with 10^4^ PFU of either wild-type, Delta (B.1.617.2) or Omicron (B.1.1.529) SARS-CoV-2 and their weight and health conditions were recorded over a period of up to 8 days. Mice infected with the wild-type virus started to show weight loss at 6 dpi and reached the humane endpoint (HEP) between 6 dpi and 7 dpi (Figure 1). Disease progression was slightly faster in mice infected with the Delta variant, which began to lose weight at 4 dpi and reach humane endpoint as early as 5 dpi. Weight loss and moderate to severe neurological signs (head tilting, poor ambulation) are the prominent criteria for the humane endpoint in mice infected with the wild-type and Delta SARS-CoV-2. In contrast, mice infected with the Omicron variant did not display any weight loss up to or past 8 dpi and invariably survived the infection with no sign of neurological symptoms.

### 3.2. Pulmonary Inflammation and Infection of K18-hACE2 Mice

**Figure 2:**
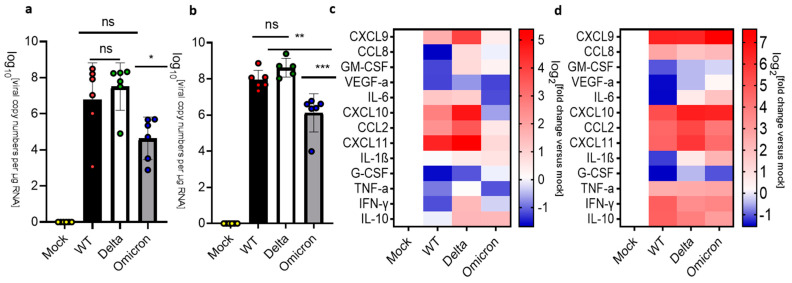
Analysis of viral RNA and cytokine/chemokine expression in the lungs of SARS-CoV-2 infected K18-hACE2. (**a**) Uninfected mice (mock) were compared against mice infected with SARS-CoV-2 wildtype (WT), Delta, and Omicron at 10^4^ PFU. Mice infected with Delta and wild type were euthanized at 3 dpi or humane endpoint (5–7 dpi). Mice infected with the Omicron variant were euthanized at 3 dpi and 8 dpi. (**a**,**b**) qPCR of viral copy numbers in the lung at 3 dpi and humane endpoint/8 dpi. Mean with SD. One-way ANOVA with Tukey’s test was performed * *p* < 0.05, ** *p* < 0.01, *** *p* < 0.001, ns: not significant (**a**) qPCR of SARS-CoV-2 viral copy numbers at 3 dpi. Mock (*n* = 4), wild type, Delta, and Omicron (*n* = 6). (**b**) qPCR of SARS-CoV-2 viral copy numbers at the humane endpoint/8 dpi. Wild type (*n* = 6), Delta (*n* = 5), and Omicron (*n* = 6). (**c**,**d**) Heatmap of cytokine and chemokine levels in the lung. The graph was plotted with the mean. The fold change was calculated using the 2^–∆∆Ct^ method and compared with mock-infected animals. The log2 (fold change) was plotted in the corresponding heat map. (**c**) Heatmap of cytokine and chemokine levels in the lung at 3 dpi Mock (*n* = 4), wild type (*n* = 6), Delta (*n* = 6), Omicron (*n* = 5). (**d**) Heatmap of cytokine and chemokine levels in the lung at the humane endpoint/8 dpi. Mock (*n* = 4), wild type (*n* = 6), Delta (*n* = 5), Omicron (*n* = 5).

Based on the differences in clinical manifestations between mice infected with the wild-type, Delta, or Omicron SARS-CoV-2, we compared the viral load and inflammation in the lung at 3 dpi versus humane endpoint. Mice infected with the Omicron variant were euthanized at day 8 dpi to make a meaningful comparison with the mice infected with wild type or Delta. At 3 dpi, the lungs of mice infected by the wild-type or Delta SARS-CoV-2 showed comparable viral loads (10^7^–10^8^ copy numbers/μg RNA), while mice infected with Omicron showed significantly lower levels of viral RNA (10^5^ copy numbers/μg RNA) (Figure 2a). Notably, infection by the Delta variant induced significantly higher levels of chemokine mRNAs such as CXCL9, CXCL10, CXCL11, and CCL2 than infection by the wild type and there was negligeable upregulation of cytokine and chemokine mRNA levels in mice infected with Omicron at 3 dpi. (Figure 2c). At the HEP, we detected higher levels of viral RNA in the lungs of mice infected with the wild type and Delta (>10^8^ copy numbers/μg RNA) than in the lungs of mice infected with Omicron (~10^6^ copy numbers/μg RNA) at 8 dpi (Figure 2b). Nevertheless, mice infected with Omicron displayed levels of inflammatory marker transcripts that were comparable to the levels observed in mice infected with wild type or Delta (Figure 2d). Thus, despite the lower levels of viral RNA, pulmonary inflammation was not attenuated in mice infected with Omicron.

### 3.3. Infection and Inflammatory Markers in the Brain of K18-hACE2 Mice

**Figure 3:**
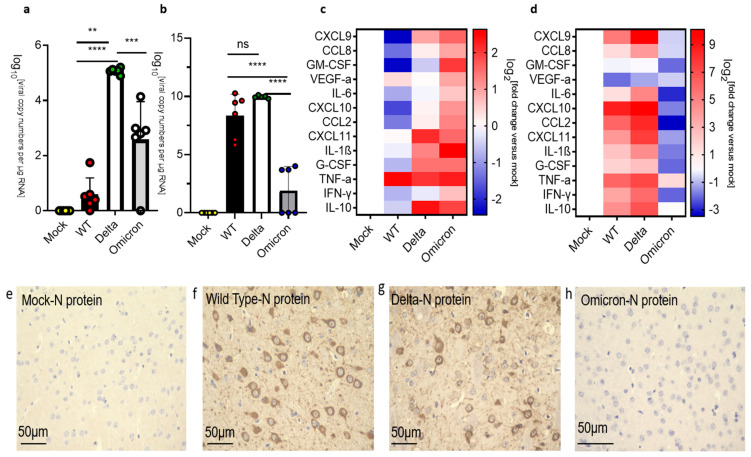
Analysis of viral RNA, viral protein, and cytokine/chemokine expression in the brains of SARS-CoV-2-infected K18-hACE2 mice. (**a**–**d**) Uninfected mice (mock) were compared against mice infected with SARS-CoV-2 wild type (WT), Delta, and Omicron at 10^4^ PFU. Mice infected with wildtype and Delta were euthanized at 3 dpi and humane endpoint (5–7 dpi), and mice infected with the Omicron variant were euthanized at 3 dpi and 8 dpi. (**a**,**b**) qPCR of viral copy numbers in the brain at 3 dpi (**a**) and humane endpoint/8 dpi. (**b**) Mean with SD. One-way ANOVA with Tukey’s test was performed. ** *p* < 0.01, *** *p* < 0.001, **** *p*< 0.0001, ns: not significant (**a**) qPCR of SARS-CoV-2 viral copy numbers at 3 dpi. Mock (*n* = 4), wild type, Delta, and Omicron (*n* = 6). (**b**) qPCR of SARS-CoV-2 viral copy numbers at the humane endpoint/8 dpi. Mock (*n* = 4), wild type (*n* = 6), Delta (*n* = 5) and Omicron (*n* = 6). (**c**,**d**) Heatmap of cytokine and chemokine levels. The graph was plotted with mean. The fold change was calculated using the 2^−∆∆Ct^ method and compared with mock-infected animals. The log2[fold change] was plotted in the corresponding heat map. (**c**) Heatmap of cytokine and chemokine levels in the brain at 3 dpi. Mock (*n* = 4), wild type, Delta, and Omicron (*n* = 6). (**d**) Heatmap of cytokine and chemokine levels in the brain at the humane endpoint/8 dpi. Mock (*n* = 4), wild type (*n* = 6), Delta (*n* = 5), Omicron (*n* = 5). (**e**) Representative images showing IHC staining for the nucleocapsid (N) protein of SARS-Co-V2 in the brain of mice infected with wild type, Delta and Omicron at HEP/8 dpi. (**e**–**h**) Immunohistochemical (IHC) staining of brains of mice infected with wild type (**f**), Delta (**g**) and Omicron (**h**). Mock infected is shown in (**e**). The photomicrographs shown are representative of the images obtained from mice infected by each SARS-Co-V2 strain. Bars: 50 μm.

**Figure 4:**
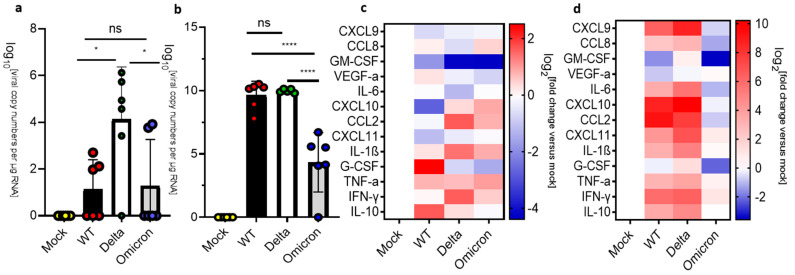
Analysis of viral RNA and inflammatory markers in the medulla oblongata of SARS-CoV-2 infected K18-hACE2 mice. Uninfected mice (mock) were compared against mice infected with SARS-CoV-2 wild-type (WT), Delta, and Omicron at 10^4^ PFU. Mice infected with Delta and wild type were euthanized 3 dpi or at the humane endpoint (5–7 dpi). Mice infected with the Omicron variant were euthanized at 3 dpi and 8 dpi. (**a**) qPCR of viral copy numbers at 3 dpi. Mean with SD. One-way ANOVA with Tukey’s test was performed * *p* < 0.01, ns: not significant mock (*n* = 4), wild type (*n* = 6), Delta (*n* = 5) and Omicron (*n* = 6) (**b**) qPCR of viral copy numbers at humane endpoint/8 dpi. Mean with SD. One-way ANOVA with Tukey’s test was performed **** *p* < 0.0001, ns: not significant. mock (*n* = 4), wild type, Delta, and Omicron (*n* = 6) (**c**) Heatmap of cytokine and chemokine levels at 3 dpi. Mock (*n* = 4), wild type, Delta, and Omicron (*n* = 6). (**d**) Heatmap of cytokine and chemokine levels at the humane endpoint/8 dpi. The graph was plotted with mean. The fold change was calculated using the 2^−∆∆Ct^ method and compared with mock-infected animals. The log2 [fold change] was plotted in the corresponding heat map. Mock (*n* = 4), wild type (*n* = 6), Delta (*n* = 5), Omicron (*n* = 6).

COVID-19 can have profound effects on the brain and SARS-CoV-2 has been detected in the human brain at autopsy [37]. Numerous studies have examined the infection of the brain by SARS-CoV2 and suggested that the virus may enter the human brain through three main routes [38,39] (Supplementary Figure S1). Here, we examined the brain of mice infected by wild type, Delta and Omicron. Note that the medulla oblongata was separated and analyzed independently of the rest of the brain (see Figure 3 and Figure 4). Mice infected with the wild-type virus did not show a significant amount of viral RNA in the brain at 3 dpi. In contrast, the brains of mice infected with the Delta virus displayed relatively high levels of viral RNA at 3 dpi (~10^5^ copy numbers/μg RNA). Interestingly, the brains of mice infected with Omicron also showed higher levels of viral RNA than mice infected with wild-type virus at 3 dpi (>10^3^ copy numbers/μg RNA) (Figure 3a). However, we did not detect SARS-Co-V2 nucleocapsid (N) protein in the brain sections of mice infected with any of these virus strains at 3 dpi by immunohistochemistry (Supplementary Figure S2). Interestingly, the analysis of cytokine and chemokine mRNAs indicated that the brains of mice infected with Omicron displayed upregulation of the inflammatory markers, while the brains of mice infected with Delta showed only increased levels of a subset of these markers, despite the high level of viral RNA at 3 dpi (Figure 3c). The brains of mice infected with the wild-type virus showed upregulation of only TNF-alpha at 3 dpi. At the HEP, mice infected with wild type and Delta showed high levels of viral RNA (10^8^–10^10^ copy numbers/μg RNA) (Figure 3b). Mice infected with wild type or Delta also showed a significant upregulation of inflammatory markers (Figure 3c). In contrast, mice infected with Omicron showed low levels of viral RNA (~10^2^–10^3^ copy numbers/μg RNA) and downregulation of inflammatory markers at 8 dpi (Figure 3d). In agreement with these results, we observed a wide distribution of SARS-Co-V2 nucleocapsid (N) protein in the brains of mice infected with wild type and Delta at the HEP, while mice infected with Omicron had no detectable virus in the brain at 8 dpi (Figure 3e–h; Supplementary Figure S3). Collectively, these data demonstrate differential levels of infection and inflammatory responses in mice infected by wild type, Delta and Omicron. 

### 3.4. Infection and Inflammation Makers in the Medulla Oblongata of K18-hACE 2 Mice

Since the human medulla oblongata has high levels of ACE2 expression [40], it represents a possible route for SARS-CoV-2 infection of the brain. We examined the medulla oblongata of mice infected with the SARS-CoV-2 strains at 3 dpi and observed significantly higher levels of viral RNA in mice infected with Delta than with either the wild type or Omicron (Figure 4a). However, infection by any of these SARS-CoV-2 strains resulted in a mild upregulation of the inflammatory markers including IL-1β, TNF-α, and IL-10 at 3 dpi (Figure 4c). At HEP, we observed a major increase in the levels of viral RNA in the medulla oblongata of mice infected with wild-type or the Delta variant (~10^10^ copy numbers/μg RNA), while a less severe increase in viral RNA levels was observed in the medulla oblongata of mice infected with the Omicron variant (~10^4^ copy numbers/μg RNA) at 8 dpi (Figure 4b). At the HEP, inflammatory markers were significantly upregulated in mice infected with wild type or Delta but downregulated in mice infected with the Omicron variant at 8 dpi (Figure 4d).

### 3.5. Infection and Inflammation Markers in the Olfactory Bulbs of K18-hACE 2 Mice

**Figure 5:**
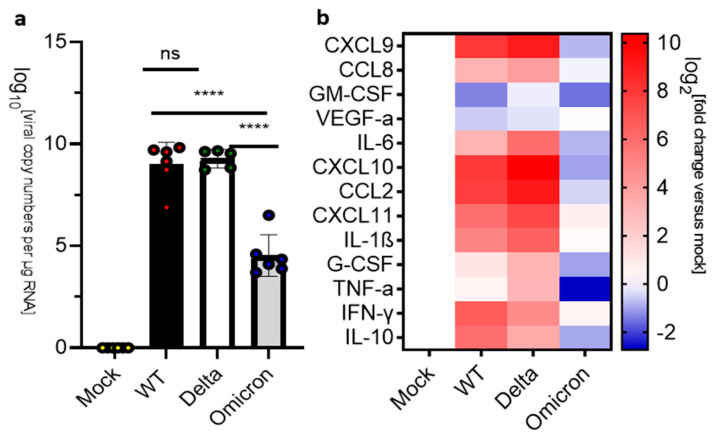
Analysis of viral RNA and inflammatory markers in the olfactory bulbs of SARS-CoV-2 infected K18-hACE2 mice at HEP/8 dpi. Uninfected mice (mock) were compared against mice infected with SARS-CoV-2 wild-type (WT), Delta, and Omicron at 10^4^ PFU. Mice infected with wild type or Delta were euthanized at the humane endpoint (5–7 dpi), while mice infected with the Omicron variant were euthanized at 8 dpi. (**a**) qPCR of viral copy numbers in the OB at the humane endpoint/8 dpi. Mean with SD. One-way ANOVA with Tukey’s test was performed **** *p* < 0.0001, ns: not significant. mock (*n* = 4), wild type (*n* = 6), Delta (*n* = 5) and Omicron (*n* = 6). (**b**) Heatmap of cytokine and chemokine levels at the humane endpoint/8 dpi. The graph was plotted with mean. The fold change was calculated using the 2^−ΔΔCt^ method and compared with mock-infected animals. The log2 [fold change] was plotted in the corresponding heat map. Mock (*n* = 4), wwild-type (*n* = 6), Delta (*n* = 5), Omicron (*n* = 6). Note: olfactory bulbs were not analyzed at 3 dpi.

The loss of smell observed in individuals infected by SARS-CoV-2 suggests that the olfactory bulb may be an important site of SARS-CoV-2 infection [41,42]. We therefore examined the levels of viral RNA and inflammatory makers in the olfactory bulbs of mice infected with wild type, Delta, and Omicron at the HEP/8 dpi. As seen in the medulla oblongata, high levels of viral RNA (~10^9^ copy numbers/μg RNA) and the upregulation of inflammatory markers were only seen in the olfactory bulbs of mice infected with wild type and Delta. The olfactory bulbs of mice infected with Omicron showed lower levels of viral RNA (~10^5^ copy numbers/μg RNA) and downregulated inflammatory markers (Figure 5a,b). 

## 4. Discussion

The neurological impact of SARS-CoV-2 has been debated since the start of the COVID-19 pandemic. Studies on the relationship between viral loads and neurological inflammation and the impact of different SARS-CoV-2 variants on the nervous system have been limited. Here, we compared the infectivity of wild-type, Delta, and Omicron SARS-CoV-2 in transgenic K18-hACE2 mice and characterized the progression of infection and inflammatory response in the lungs and brains of these animals. We observed that transgenic mice infected with Omicron displayed minimal weight loss and reduced disease progression as compared to mice infected with the wild type and Delta variant, which is in line with infection studies in hamsters [16,43,44]. Mice infected with the Delta variant had higher viral RNA levels and high levels of expression of inflammatory markers at the humane endpoint in the lung, suggesting that this SARS-CoV-2 variant results in enhanced lung infection and cellular damage. The high levels of lung inflammation caused by the Delta variant were also observed in other studies using the K18-hACE2 mouse model [45,46]. Interestingly, although viral RNA levels in the lungs of Omicron-infected mice were significantly lower, infection by this variant resulted in robust upregulation of inflammatory markers at both 3 dpi and 8 dpi as compared to wild type and Delta at 3 dpi and HEP. As increased expression of proinflammatory cytokines has been reported to be related to reduced lung function and tissue damage in both acute and post-acute infections [45,46,47], the high expression levels of cytokine/chemokine genes we observed in the lungs of mice infected by either wild type, Delta, or Omicron is likely to cause lung tissue damage and reduced lung function in post-acute infections.

Many studies have shown significant and long-lasting neurological manifestations of SARS-CoV-2 infection [38]. Our analysis of the brains of mice infected with the Omicron variant shows reduced viral RNA levels as compared to the brains of mice infected with wild type or Delta at the humane endpoint/8 dpi. We did not examine how the virus reached the brain and whether it directly infected neurons or other brain cell types. However, studies by other groups have provided evidence of viral entry into the brain via distinct ways as well as direct infection of neurons in K19-hACE2 mice [48,49,50]. Regardless of the mechanism of entry, our study demonstrates that infection by different SARS-CoV-2 strains elicits distinct transcriptional responses by inflammatory markers in the brains of k18-hACE2 mice. The brains of mice infected with Omicron showed a significant upregulation of cytokines and chemokines at 3 dpi. This increase appears to be transient, as these markers were all downregulated at 8 dpi, suggesting that infection by this variant results in attenuated neurological consequences compared to mice infected with either wild type or Delta. This outcome agreed with the reported lack of neurological symptoms after the Omicron outbreak in human cohort studies [51,52]. 

We observed viral RNA and a mild upregulation of inflammatory markers in the medulla oblongata of mice infected with wild type, Delta, and Omicron at 3 dpi. This is consistent with the detection of the virus and the activation of immune response cells in the brainstems of COVID-19 patients [30]. However, while the high levels of viral RNA and a significant upregulation of inflammatory genes were observed in mice infected with wild type and Delta, mice infected with the Omicron variant showed lower levels of viral RNA and the downregulation of inflammatory genes at 8 dpi. Lower levels of viral RNA and the downregulation of inflammatory markers were also seen in the olfactory bulbs of mice infected with Omicron at 8 dpi as compared to Delta and Omicron at the humane endpoint; a result that is similar to the low viral load observed in the olfactory bulbs of hamsters infected with Omicron [53]. Moreover, in contrast to mice infected with wild type or Delta, the infection by Omicron did not result in any upregulation of inflammatory markers in the olfactory bulbs. These findings align with cohort studies that reported limited loss of smell and taste in patients infected with the Omicron variant [54,55]. 

## 5. Conclusions

Collectively, these data demonstrate that K18-hACE2 mice infected with wild type, Delta, and Omicron showed distinct levels of viral RNA and inflammatory responses in the lungs, brain, medulla oblongata and olfactory bulbs during the infection. Importantly, the inflammatory response in the brains of mice infected by the Omicron variant was limited to the early phase of infection and did not lead to health conditions that required euthanasia. 

### Limitations of the Study

The study has potential limitations. The transgenic K18-hACE2 mouse model is highly susceptible to the lethality of SARS-CoV-2 infection and does not fully mirror the infection in humans. This model is therefore not suitable for studying the long-term effects of SARS-CoV-2 infection (long COVID). Nevertheless, our data show that K18-hACE2 mice infected with the Omicron variant recovered from the infection, suggesting that infection of K18-hACE2 mice by this variant may offer a model system for the study of long COVID. Another limitation of the study is in the use of RT-qPCR to measure changes in cytokine and chemokine mRNAs. While these transcriptional events are likely associated with changes in protein levels, we have not performed experiments to assess these changes. 

## Data Availability

Data are available from corresponding authors upon request.

## Supplementary Materials

The following supporting information can be downloaded at: https://www.mdpi.com/article/10.3390/v16010060/s1, Figure S1: Schematic representation of mouse brain with possible routes of CNS infection by SARS-CoV-2; Figure S2: Representative images showing IHC staining for the nucleocapsid (N) protein of SARS-Co-V2 in the brain of mice infected with Wild-type, Delta and Omicron at 3 dpi; Figure S3: Representative images showing IHC staining for the nucleocapsid (N) protein of SARS-Co-V2 in the brain of mice infected with Wild-type, Delta and Omicron at HEP/8 dpi.
