# Supplementary material for: Differential Outcomes of Infection by Wild-Type SARS-CoV-2 and the B.1.617.2 and B.1.1.529 Variants of Concern in K18-hACE2 Transgenic Mice
Source: Viruses. 2023 Dec 29;16(1):60. doi: 10.3390/v16010060 (PMC10820160; doi:10.3390/v16010060)
Supplement: Supplementary file 1 [file viruses-16-00060-s001.zip › viruses-2784592-SI.pdf]

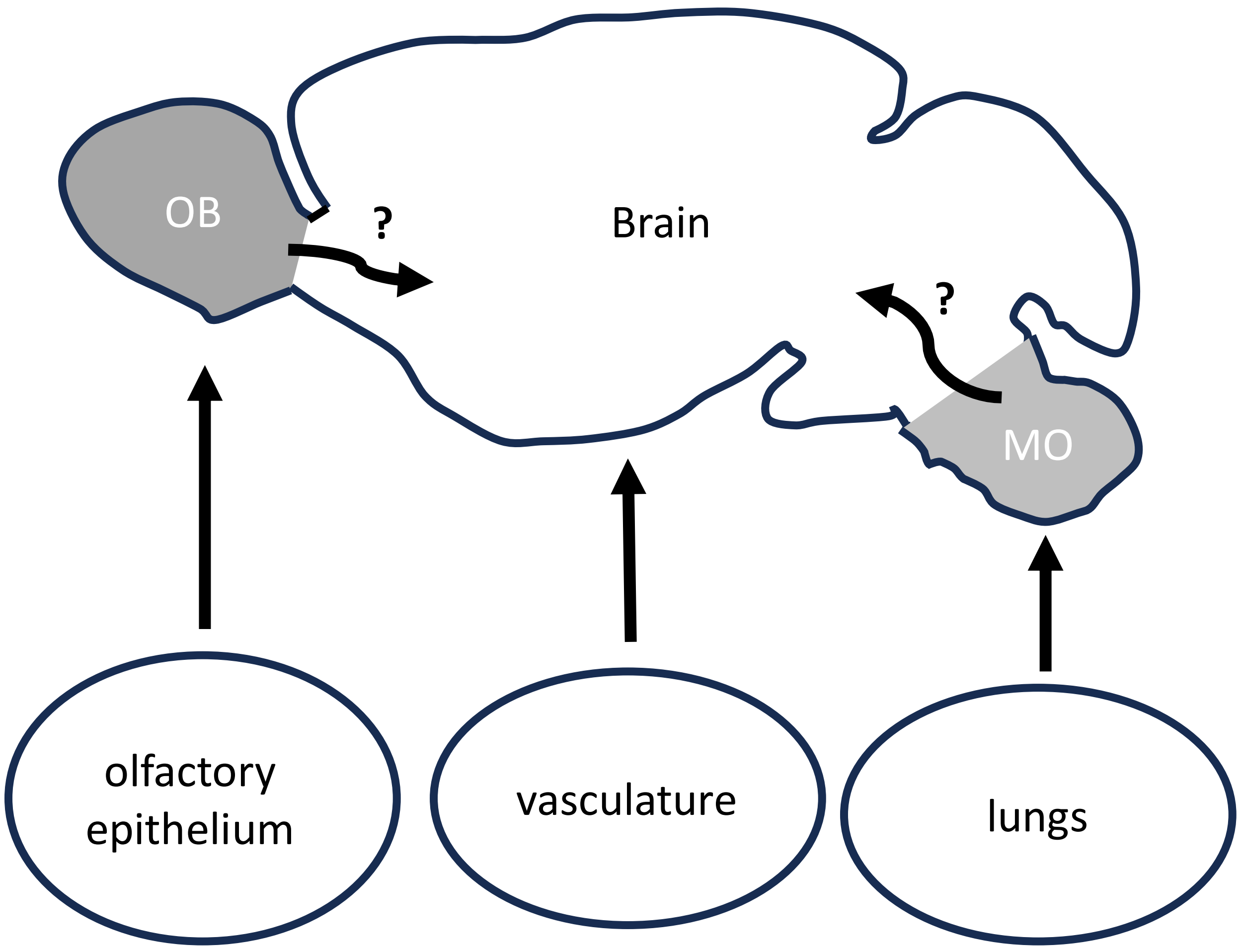

Supplementary Figure S1: Schematic representation of mouse brain with possible routes of CNS infection by SARS-CoV-2. 1. Olfactory route: 2. Vasculature and crossing the blood-brain barrier; 3. Travels from the lungs to the medulla oblongata brain stem through the vagal nerve. OB: olfactory bulb; MO: medulla oblongata

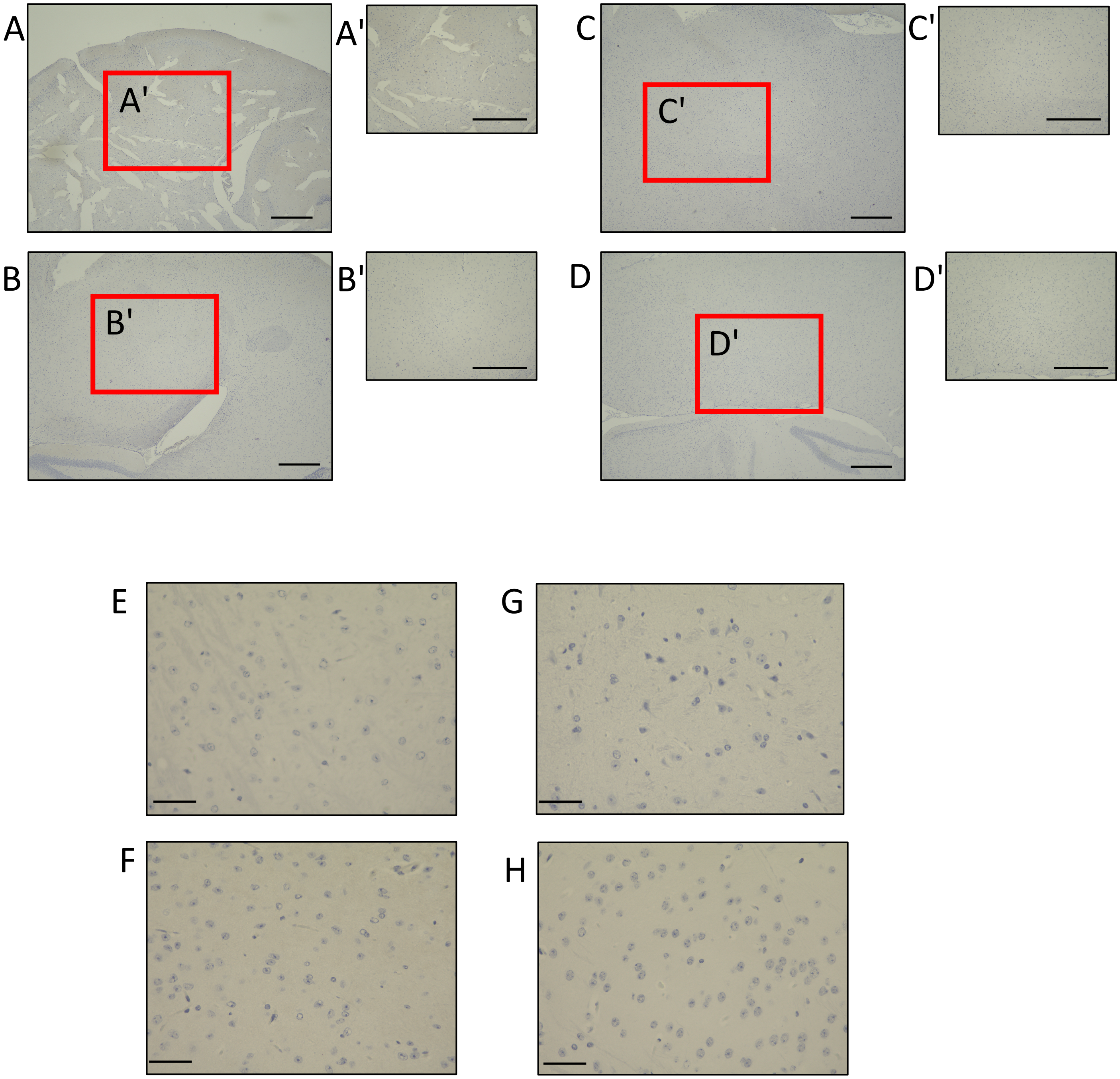

Supplementary Figure S2: Representative images showing IHC staining for the nucleocapsid (N) protein of SARS-Co-V2 in the brain of mice infected with wild-type, Delta and Omicron at 3dpi.
Top panels (A-D): Immunohistochemical (IHC) staining of brains of mice infected with wild-type (B), Delta (C) and Omicron (D). Panels A'-G' show images of inset in panels A-D at 10x magnification. Mock control brain is shown in panel A. Bars: 500 µm.
Bottom panels (E-H): Higher magnification (40x) images of brain sections of mice infected with wild-type (F), Delta (G) and Omicron (H) at 3dpi. Mock control brain is shown in panel E.
Bars: 50 µm

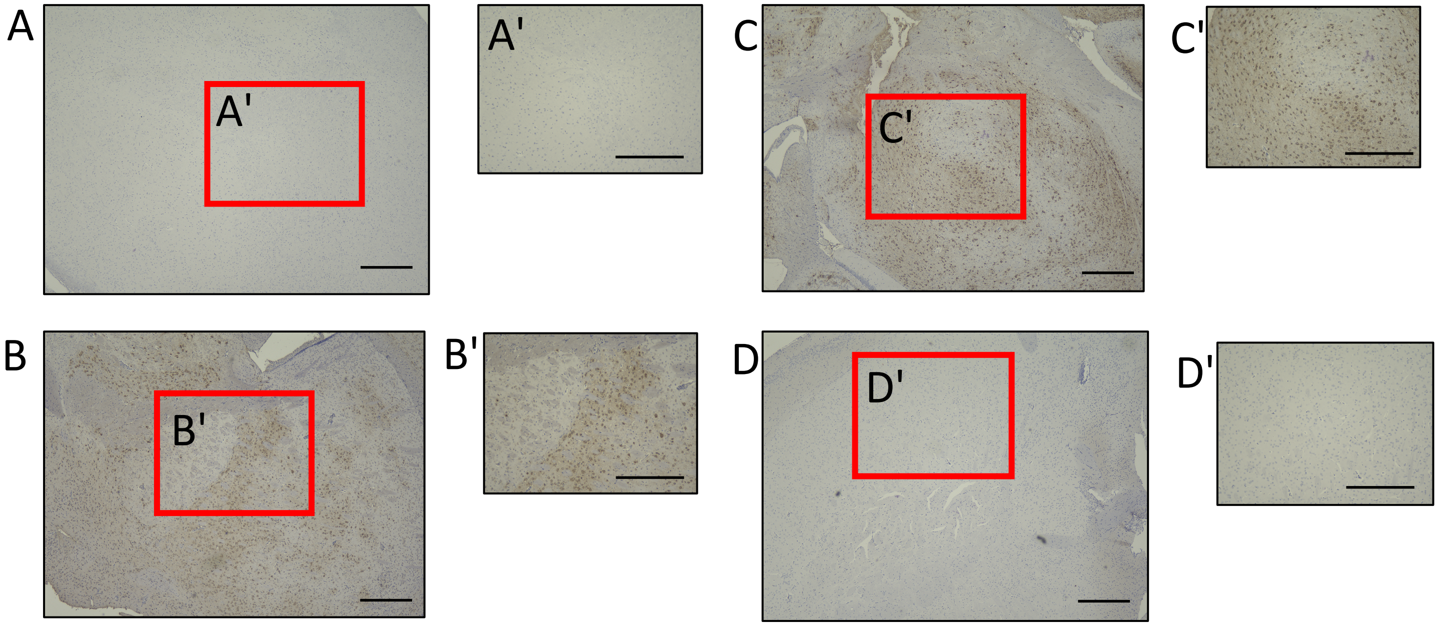

Supplementary Figure S3: Representative images showing IHC staining for the nucleocapsid (N) protein of SARS-Co-V2 in the brain of mice infected with wild-type, Delta and Omicron at HEP/8dpi. Immunohistochemical (IHC) staining of brains of mice infected with wild-type (B), Delta (C) and Omicron (D). Mock control brain is shown in panel A. Panels A'-G' show higher magnification (10X) images of inset in panels A-G. Only brain sections of mice infected with wild-type (panel B) and Delta (panel C) show N protein staining. and Bars: 500 µm.
